# The association of mindfulness on psychological distress among Chinese university students: a culturally contextualized investigation

**DOI:** 10.3389/fpubh.2026.1828163

**Published:** 2026-06-23

**Authors:** Tao Liu, WenJing Zhang, Rong Fu

**Affiliations:** 1 Academy of the Zhouhuaminzu Community, Lishui University, Lishui, China; 2 Department of Sociology, School of Law, Hangzhou Dianzi University, Hangzhou, China; 3 College of Economics, Hangzhou Dianzi University, Hangzhou, China

**Keywords:** cognitive reappraisal, expressive suppression, mindfulness, psychological distress, resilience, social support

## Abstract

**Introduction:**

Mindfulness training benefits psychological well‑being, but its mechanisms in culturally specific contexts (e.g., Chinese university students facing academic pressure and family expectations) remain poorly understood.

**Methods:**

Cross‑sectional stratified random sampling of 432 undergraduates from four Chinese universities. Standardized scales measured mindfulness, psychological distress, resilience, emotion regulation (cognitive reappraisal, expressive suppression), social support (instrumental, emotional, informational), academic pressure, and family expectations. Analysis used hierarchical regression, SEM, and moderated mediation.

**Results:**

Mindfulness negatively correlated with psychological distress (*β* = –0.34, *p* < 0.001). This correlation was moderated by academic pressure (weaker under high pressure) and family expectations (stronger under high expectation). Resilience and cognitive reappraisal showed synergistic mediation (total *β* = 0.71). Effects varied by gender and academic discipline.

**Discussion:**

The mindfulness‑distress relationship is culturally contingent. Interventions should be locally tailored, combining personal training with environmental support.

## 1 Introduction

Mental health has become a critical global public health issue. As a fundamental element of well-being, mental health represents an individual’s dynamic balance across cognitive, emotional, and behavioral aspects (1). Emerging adulthood, which is a high risk period for psychological disorders (2), presents distinct challenges for university students as they navigate academic transitions and changing societal roles. In China, recent research emphasizes the gravity and cultural distinctiveness of these challenges. A meta-analysis published in BMC Psychology in 2025 reported that the combined prevalence rate of depression among Chinese university students was 34.70%, suggesting a high probability of psychological distress within this group (3). The 2023 *Blue Book of Chinese University Students’ Mental Health* surveyed 46,582 undergraduates from 127 institutions, revealing a 31.2% depression rate (4), a 6.5% increase compared to the 2019 baseline data (5). Academic pressure and intergenerational conflicts stemming from family expectations were identified as key influencing factors: students who studied for over 10 h per day had a 39.7% depression rate, while those experiencing academic conflicts related to family faced a 187% higher risk of depression (6). China’s depression rate (31.2%) is higher than those in Southeast Asia (Philippines: 27.1%, Malaysia: 25.9%) (7) and the WHO global average (23.8%) (8). This disparity is likely attributable to structural stressors in China’s education system, such as grade competition and the pressure of postgraduate admissions. Culturally, Chinese society is characterized by collectivist values that prioritize group harmony, family reputation, and social conformity over individual autonomy (9). In this cultural context, individuals define themselves in relation to significant others, including parents, extended family, and the broader community, rather than as independent entities (10). Consequently, academic achievement in China holds moral and relational significance: academic success brings honor to the family, while failure may be regarded as a source of shame not only for the individual but also for the entire family lineage. This phenomenon, commonly referred to as “face” (mianzi) in Chinese cultural discourse, reflects an individual’s social status and the respect accorded by others, and it exacerbates the pressure to meet intergenerational expectations (11, 12). Therefore, academic pressure and family expectations in China are not merely individual stress factors but culturally ingrained demands with collective implications. Understanding how mindfulness functions under such culturally specific circumstances requires transcending universal assumptions and examining how collectivist norms moderate the transformation of mindfulness skills into psychological outcomes. These findings highlight the necessity for culturally informed intervention models to address systemic challenges. In the present study, we specifically focus on psychological distress, as measured by the SCL-90, rather than positive mental health, in order to capture the clinically relevant symptoms prevalent in this population.

Mindfulness, a practice that emphasizes present moment awareness and nonjudgmental acceptance (13), has been shown to be associated with the alleviation of depression and anxiety (14), in part through the reduction of rumination (15). Neuroscience research indicates that mindfulness enhances emotion regulation by modifying prefrontal limbic connectivity. For example, Tang et al. (16) discovered that mindfulness meditation leads to an increase in the thickness of the prefrontal cortex and suppresses the activity of the default mode network, thereby providing a neurobiological foundation for cognitive reappraisal. Hölzel et al. (17) further identified dual mechanisms: “top down” cognitive control (e.g., activation of the dorsolateral prefrontal cortex) and “bottom up” bodily awareness (e.g., enhanced function of the insula). These findings support the integrated pathways that connect mindfulness to psychological resilience and adaptive emotion regulation.

Although studies have confirmed that mindfulness is associated with reduced psychological distress through resilience (18) or cognitive reappraisal (19), three limitations still exist. Firstly, resilience and emotion regulation are frequently examined independently (20), disregarding their potential interactions. For instance, resilience may enhance the efficacy of reappraisal, while excessive expressive suppression may impede resilience. Secondly, social support is generally regarded as a unified construct, overlooking the distinct effects of instrumental, emotional, and informational support (21). Thirdly, few studies explore how academic and familial pressures in China restric mindfulness interventions, which limits cross cultural applicability. Neuroimaging comparisons disclose cultural differences: Western practitioners rely more on prefrontal analytical processing, whereas Eastern practitioners engage holistic default mode networks (22). Chen et al. (23) point out that collectivist values such as “face-saving” and family obligations may undermine nonjudgmental acceptance in mindfulness training, highlighting the crucial role of cultural context.

**Figure 1:**
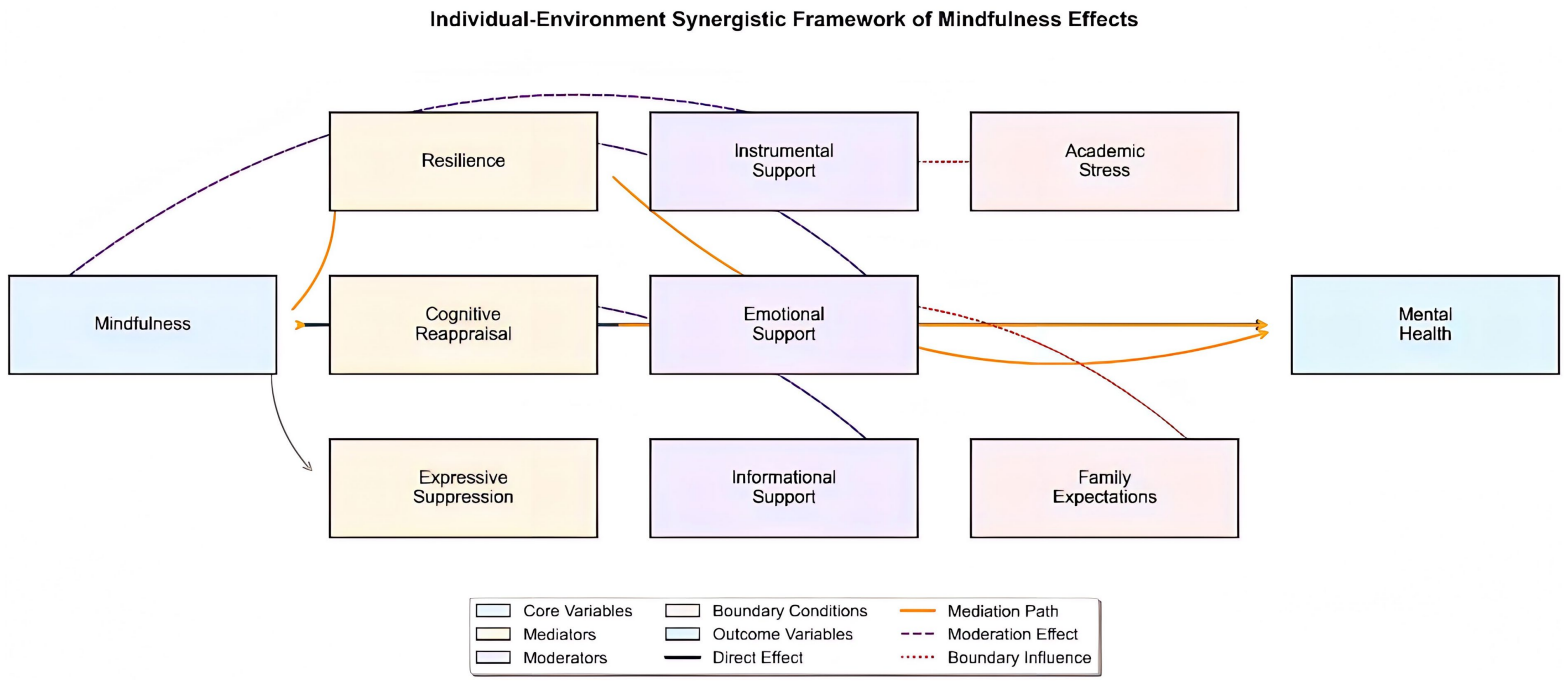
Hypothesized research model.

Rooted in the Conservation of Resources Theory (24) and Social Cognitive Theory (25), this study puts forward an individual and environment framework (Figure 1) to investigate the mechanisms of mindfulness. Four research questions direct the exploration: (1) Does mindfulness exert direct effects on psychological distress when cultural stressors are taken into account? (2) Do resilience and emotion regulation strategies serve as complementary or competing mediators? (3) How do different types of social support differentially moderate the pathways of mindfulness? (4) Do academic pressure and family expectations represent cultural boundary conditions for mindfulness interventions?

To establish a unified basis, this study employs the Conservation of Resources (COR) theory (24) as its overarching framework. The COR Theory posits that individuals endeavor to safeguard valuable resources; distress emerges when resources are lost or threatened. Mindfulness is a personal resource that aids in preventING depletion and promotes gain. Social support (instrumental, emotional, informational) offers external resources. Cultural stressors (academic pressure and family expectations) function as boundary conditions that either deplete or enhance the availability of resources.

The Social Cognitive Theory (25) complements this perspective: individuals with higher levels of mindfulness and support are more prone to manage stressors in an adaptive manner. The emotion regulation model (26) delineates the pathways: promoting reappraisal while reducing suppression. This framework facilitates the study of synergy (resilience and reappraisal) and competition (reappraisal and suppression) across different cultures.

Exploratory analysis of gender differences. Although this study is not primarily aimed at an investigation centered on gender, a substantial amount of literature on gender disparities in emotion regulation and social support [e.g., socioemotional selectivity theory (27)] suggests that the efficacy of certain pathways, especially the impact of emotional support on resilience, may vary across genders. Consequently, we examine whether the moderating function of emotional support differs between female and male students. These analyses are exploratory in essence, and the findings should be regarded as generating hypotheses rather than confirmatory. Prudent interpretation is particularly necessary for subgroup comparisons involving small effect sizes or confidence intervals that encompass values close to zero.

Current research reveals critical gaps. While Su et al. (28) established a link between mindfulness and reduced depression through resilience, they were unable to account for why some resilient individuals still encounter challenges in emotion regulation, suggesting the existence of unobserved mediating mechanisms. Liu et al. (29) identified cognitive reappraisal as a mediator but reported a lower explanatory power (*R*^2^ = 0.31) compared to Western studies (*R*^2^ = 0.48), indicating potential cultural moderators. Pan (30) discovered that instrumental support influences mindfulness adherence in Chinese students but did not explore its dynamic role among intervention outcomes.

This research puts forward seven hypotheses to fill these gaps: Hypothesis 1 challenges the universality of mindfulness effects by introducing academic and familial pressures as cultural moderators. Hypotheses 2 and 3 propose a synergistic resilience and reappraisal model while testing competing suppression effects. Hypotheses 4, 5 and 6 distinguish how instrumental, emotional, and informational support uniquely moderate mindfulness pathways. Hypothesis 7 establishes a hierarchical model in which academic and familial pressures secondarily moderate social support effects.

The framework presents methodological and practical advancements. Employing moderated mediation models with longitudinal and multi group analyses contributes to reducing bias stemming from reverse causality in cross-sectional studies. Practically, if Hypothesis 5 validates the gender specific benefits of emotion focused resilience pathways, targeted interventions may be developed. For instance, integrating time management tools (Hypothesis 4) for STEM students or combining cognitive reappraisal with family communication training (Hypothesis 6).

This study elucidates how mindfulness operates under different conditions by integrating stress buffering and resource gain theories. While previous research emphasizes individual resource accumulation (e.g., resilience), this study demonstrates that when environmental stressors surpass critical thresholds (Hypothesis 7), social support buffers may become ineffective, highlighting the necessity for systemic institutional reforms in addition to individual interventions.

This multi level model promotes theoretical progress by shifting the focus from addressing individual problems to enhancing the interaction between individuals and their environment. The findings will provide insights for China’s mental health policies, especially in strengthening campus support systems and managing structural stressors.

## 2 Literature and hypothesis

### 2.1 Direct effects of mindfulness on psychological distress and cultural moderation

Mindfulness, a practice originating from Buddhist traditions and adapted by modern psychology, emphasizes nonjudgmental awareness of present moment experiences (13). Empirical research has demonstrated its effectiveness in alleviating psychological distress. For example, Hofmann et al. (14) discovered through meta-analyses that mindfulness training can reduce anxiety and depression while enhancing life satisfaction. This effect may be attributed to the increased awareness of thoughts and emotions, which disrupts cycles of negative thinking and reduces rumination (15).

However, among Chinese university students, cultural factors uniquely influence the outcomes of mindfulness. Two prominent stressors in China’s education system, namely academic pressure and familial expectations, moderate these effects.

The intense competition for grades and postgraduate placements (4) depletes psychological resources, limiting students’ ability to apply mindfulness skills. Within the framework of the Conservation of Resources (COR) theory, chronic academic stress depletes mental reserves, thus diminishing the buffering effects of mindfulness (24).

Moderate family expectations can motivate achievement (31), which is in line with collectivist values such as “honoring ancestors.” Such expectations may synergize with mindfulness by fostering adaptive psychological responses. For instance, family driven achievement goals could enhance the protective effects of mindfulness when they are aligned with personal growth.

Hypothesis 1

H1a: Mindfulness is negatively associated with psychological distress.

H1b: Academic pressure negatively moderates the association between mindfulness and psychological distress.

H1c: Family expectations positively moderate the association between mindfulness and psychological distress.

### 2.2 Mediating pathways: psychological resilience and emotion regulation

#### 2.2.1 Complementary mediation: synergy between resilience and cognitive reappraisal

Psychological resilience, defined as the dynamic capacity to adapt under stress (32), emerges from interactions between personal resources (e.g., self efficacy) and environmental support (24). Mindfulness enhances resilience by promoting present moment awareness, enabling individuals to derive growth from adversity (33). Resilient individuals can navigate challenges more effectively and recover more rapidly from setbacks.

Cognitive reappraisal reduces emotional arousal by reframing stressors (26). Resilience and reappraisal reinforce each other: resilient individuals reframe failures as challenges (20), and reappraisal conserves resources and strengthens resilience (19).

Mindfulness likely reduces psychological distress through complementary mediation: it enhances resilience, which promotes reappraisal, while reappraisal reciprocally strengthens resilience. This bidirectional relationship suggests a synergistic pathway.

Hypothesis 2

H2a: Psychological resilience partially mediates the association between mindfulness and psychological distress.

H2b: Cognitive reappraisal partially mediates the association between mindfulness and psychological distress.

H2c: Psychological resilience and cognitive reappraisal exhibit a synergistic mediating effect.

#### 2.2.2 Competing mediation: cognitive reappraisal vs. expressive suppression

Expressive suppression, a maladaptive strategy involving the deliberate inhibition of emotional expression (26), impairs psychological distress. Chronic suppression depletes cognitive resources (34), weakens resilience (35), and correlates with depression in collectivist cultures where “face-saving” norms are prevalent (36).

In contrast, mindfulness promotes cognitive reappraisal. By enhancing emotional awareness, mindfulness enables early and objective evaluation of emotional triggers, facilitating reappraisal. Wang et al. (37) observed an 18% decline in suppression and a 34% increase in reappraisal after a 8 week mindfulness training, which was linked to functional changes in the anterior cingulate and dorsolateral prefrontal cortex (38).

Thus, mindfulness reduces psychological distress by prioritizing reappraisal over suppression. The role of suppression as a mediator is negligible, reflecting the strategic competition between regulation approaches.

Hypothesis 3

H3a: Cognitive reappraisal is a significant mediator of the association between mindfulness and psychological distress.

H3b: Expressive suppression is not a significant mediator of the association between mindfulness and psychological distress.

Unlike H2, which focuses on synergy among adaptive pathways, H3 examines the relative dominance of adaptive over maladaptive emotion regulation.

Together, H2 and H3 capture two distinct dimensions of mindfulness pathways: the complementary interplay of resilience and reappraisal, and the competitive advantage of reappraisal over suppression. These dimensions are theoretically compatible rather than contradictory.

### 2.3 Social support as a moderator

Social support, defined as the perceived care, respect, and sense of belonging within a social network (39), varies in type and function. Social support is commonly classified into three types: instrumental, emotional, and informational support (40), each with distinct moderating effects.

#### 2.3.1 Instrumental support’s direct reinforcement

Instrumental support (e.g., academic guidance, time management tools) reduces objective stressors (41). For high pressure groups such as STEM students, such support complements mindfulness by improving resource allocation. For example, study aids alleviate academic demands, freeing cognitive resources for mindfulness practice. Such support may enhance self efficacy, increasing confidence in applying mindfulness under stress (25).

Hypothesis 4

H4a: Instrumental support positively moderates the direct association between mindfulness and psychological distress.

H4b: The moderating effect of instrumental support is stronger among students with high academic pressure than among those with low academic pressure.

#### 2.3.2 Emotional support’s mediation enhancement

Emotional support, which provides psychological comfort and validation (21), strengthens resilience by fulfilling the need for belonging. Within the COR framework, emotional support can be regarded as an external resource that is particularly accessible to women. Consistent with the socioemotional selectivity theory (27), women tend to prioritize emotional bonds under stress, making this support particularly impactful for them. Mindfulness may heighten awareness of available emotional support, amplifying its resilience building effects.

Hypothesis 5

H5a: Emotional support enhances the mediating role of psychological resilience in the association between mindfulness and distress.

H5b: This enhancement effect is stronger among female students than among male students (exploratory hypothesis).

#### 2.3.3 Informational support’s cognitive reappraisal boost

Informational support (e.g., stress management skills, career guidance) reduces cognitive load and improves the efficacy of reappraisal (27). For students under high familial expectations, structured information helps them navigate social comparison pressures (31). Mindfulness may optimize the integration of such support, enhancing the mediating role of reappraisal.

Hypothesis 6

H6a: Informational support enhances the mediating role of cognitive reappraisal in the association between mindfulness and distress.

H6b: This enhancement effect is stronger among students with high family expectations than among those with low family expectations.

### 2.4 Boundary effects of cultural stressors

Academic pressure and familial expectations act as cultural boundary conditions. Excessive academic stress causes cognitive overload (42), rendering instrumental support ineffective even when available. Conversely, strong familial expectations may deepen emotional bonds (24), amplifying the benefits of emotional support. For example, family encouragement under high expectations can enhance resilience during stress.

Within this framework, academic pressure in China is amplified by the cultural belief that individual success or failure reflects on the entire family, a phenomenon known as “relational interdependence” (10). Conversely, family expectations, when perceived as supportive rather than controlling, may activate achievement motivation in a culturally congruent manner (43). Accordingly, it is hypothesized that the effect of mindfulness are moderated by these culturally specific stressors, with the directionality depending on whether the cultural demand depletes psychological resources (academic pressure) or aligns with culturally normative sources of meaning (family expectations).

Hypothesis 7

H7a: Academic pressure weakens the moderating benefit of instrumental support.

H7b: Family expectations strengthen the moderating benefit of emotional support on resilience.

This study proposes a dual dimensional mediation model. The first dimension (H2) pertains to the synergy among adaptive resources; it is hypothesized that psychological resilience and cognitive reappraisal reinforce each other, jointly transmitting the benefits of mindfulness. The second dimension (H3) concerns competition between adaptive (reappraisal) and maladaptive (expressive suppression) strategies, hypothesizing that mindfulness selectively activates reappraisal while deactivating suppression. These two dimensions are not mutually exclusive; rather, they reflect different layers of psychological functioning (resource accumulation vs. strategy selection). The overall model is therefore both complementary (among adaptive pathways) and competitive (across adaptive vs. maladaptive pathways).

## 3 Data, variables, and methods

### 3.1 Data

This cross-sectional study adopted stratified random sampling across four universities located in the eastern (Beijing, Shanghai), central (Wuhan), and western (Chengdu) regions of China. Sampling procedure. A stratified random sampling design was employed. Strata were defined based on two criteria: (1) geographical region (eastern, central, and western China) and (2) university type (comprehensive universities versus science engineering focused institutions). Within each of the six resulting strata (3 regions × 2 types), one university was randomly selected from the pool of accredited universities within that stratum. After consolidating overlapping categories, this process resulted in a total of four universities (eastern region: one comprehensive and one science engineering university; central region: one comprehensive university; western region: one comprehensive university).

Within each selected university, student sampling was carried out as follows: First, academic year (freshman, sophomore, junior, senior) and major category (STEM, humanities, others) were employed as additional stratification variables to guarantee proportional representation. Second, 3–5 classes were randomly selected per stratum (i.e., per combination of year and major category) based on the class rosters provided by the university administration. All students present in the selected classes were invited to participate.

Data (Table 1) were collected via Wenjuanxing, an online survey platform, encompassing humanities, STEM, and other disciplines. From 504 initial responses, 432 valid samples were retained after excluding incomplete or inconsistent responses (85.7% validity rate). The sample size met the requirements of structural equation modeling (SEM; 30 parameters; 14.4:1 case to parameter ratio). Ethical approval was obtained, with informed consent and anonymity ensured.

**Table 1:** Demographic characteristics of the valid sample (*N* = 432).

Variable	Category	Frequency	Percentage (%)
Gender	Male	198	45.8
	Female	234	54.2
Age	18–20 years	167	38.7
	21–23 years	231	53.5
	≥24 years	34	7.8
Grade	Freshman	112	25.9
	Sophomore	135	31.3
	Junior	126	29.2
	Senior	59	13.6
Major	Liberal Arts	148	34.3
	STEM	227	52.5
	Other	57	13.2
Family economic status	Low-income	89	20.6
	Middle-income	287	66.4
	High-income	56	13.0
Total		432	100

### 3.2 Variables

#### 3.2.1 Dependent variable

Psychological Distress: Measured using the SCL-90 scale (44), which consists of 90 items and assesses psychological symptoms across cognitive, emotional, and behavioral domains. Items are rated from 0 to 4 (0 = never; 4 = severe). Higher scores indicate greater psychological distress. The 9 factor structure demonstrated good fit: *χ*^2^/df = 2.85, RMSEA = 0.071, CFI = 0.952, TLI = 0.941. Excellent reliability (*α* = 0.971).

#### 3.2.2 Independent variable

Mindfulness: Assessed via the validated Chinese Five Facet Mindfulness Questionnaire (45). The scale, which consists of 39 items (5 factors: Observe, Describe, Act with Awareness, Non Judge, Non React), showed a strong fit (*χ*^2^/df = 2.31, RMSEA = 0.063, CFI = 0.981) and reliability (*α* = 0.892 overall; 0.79 to 0.88 subscales). Responses range from 0 to 4 (higher scores indicate greater mindfulness).

#### 3.2.3 Mediating variables

Psychological Resilience: Measured using a subscale consisting of 7 items from Zhang’s Psychological Capital Scale (46). The single factor model showed excellent fit (*χ*^2^/df = 1.76, RMSEA = 0.048) and reliability (*α* = 0.823). Reverse scored items were adjusted.

Emotion Regulation: Assessed via Wang’s scale consisting of 14 items (47) with two subscales: cognitive reappraisal (adaptive) and expressive suppression (maladaptive). The two factor model fit well (*χ*^2^/df = 2.47, RMSEA = 0.068), with α = 0.847 overall (reappraisal: 0.82; suppression: 0.79). Higher suppression scores indicate poorer regulation.

#### 3.2.4 Moderating variables

Social Support: Barrera’s Social Support Behaviors Scale (48), which consists of 40 items, was used to assess three types: emotional, instrumental, and informational. The three factor model demonstrated a good fit (*χ*^2^/df = 2.58, RMSEA = 0.074) and high reliability (*α* = 0.926 overall; 0.85 to 0.91 subscales).

Academic Stress: Li and Mei’s scale (49), which consists of 31 items, measured three domains: academic demands, personal conflicts, and negative life events. The three factor model fit well (*χ*^2^/df = 2.39, RMSEA = 0.072), with *α* = 0.908 overall.

Family Expectations: A culturally adapted scale consisting of 12 items (31) was used to assess four domains: economic support, social status, academic/career achievement, and marital timelines (Table 2). The four factor model showed an acceptable fit (*χ*^2^/df = 2.50, RMSEA = 0.079) and reliability (*α* = 0.876).

**Table 2:** Chinese college students’ family expectations scale.

Dimension	Item	Response Options
Economic expectations	1. My family explicitly expects me to contribute financially to the household after employment.	Strongly disagree, Disagree, Neutral, Agree, Strongly agree
	2. I would feel guilty if I could not financially support my family.	Strongly disagree, Disagree, Neutral, Agree, Strongly agree
	3. My family expects me to use my income to improve their living conditions (e.g., purchasing a house, covering medical expenses).	Strongly disagree, Disagree, Neutral, Agree, Strongly agree
Social expectations	4. My family frequently compares my achievements with those of relatives’ children.	Strongly disagree, Disagree, Neutral, Agree, Strongly agree
	5. If my career has low social status, my family would feel it “loses face.”	Strongly disagree, Disagree, Neutral, Agree, Strongly agree
	6. My family monitors whether my social media image aligns with the “ideal child” persona.	Strongly disagree, Disagree, Neutral, Agree, Strongly agree
Academic and career expectations	7. My parents have set specific educational goals for me (e.g., obtaining a master’s/doctoral degree).	Strongly disagree, Disagree, Neutral, Agree, Strongly agree
	8. My family opposes me choosing interest-driven but financially unstable careers.	Strongly disagree, Disagree, Neutral, Agree, Strongly agree
	9. My family expects me to work in large internet companies or state-owned enterprises (SOEs).	Strongly disagree, Disagree, Neutral, Agree, Strongly agree
Marital expectations	10. My family believes I must marry before a certain age (e.g., 30 years old).	Strongly disagree, Disagree, Neutral, Agree, Strongly agree
	11. My family interferes with my criteria for choosing a partner (e.g., education level, household registration, income).	Strongly disagree, Disagree, Neutral, Agree, Strongly agree
	12. Remaining unmarried would be seen as shirking family responsibilities.	Strongly disagree, Disagree, Neutral, Agree, Strongly agree

#### 3.2.5 Control variables

Control variables included gender, age, academic year, major, and household income.

### 3.3 Methods

To conduct a systematic exploration of the multilevel mechanisms by which mindfulness is associated with the psychological distress of university students, this study adopted a comprehensive analytical approach.

Data preparation entailed strict quality control measures. Cronbach’s alpha (*α* > 0.7) and composite reliability (CR > 0.6) were employed to evaluate the scale reliability of the scale, while confirmatory factor analysis (CFA) was utilized to assess the structural validity (RMSEA < 0.08, CFI/TLI > 0.90). Common method bias was controlled through Harman’s single factor test, guaranteeing that the first factor accounted for less than 40% of the variance. Missing data were handled using full information maximum likelihood (FIML) estimation, and continuous variables were standardized (z scores) to eliminate scale differences.

Hypothesis testing employed multiple methodologies. For H1 (direct effects and cultural moderation), hierarchical regression analysis in SPSS (a three step model) was used to examine control variables, mindfulness, and interaction terms. Hypotheses H2 and H3 (mediating pathways) were tested using structural equation modeling (SEM) and bootstrapped mediation analysis (PROCESS Model 4) to compare the complementary or competing effects of resilience and emotion regulation. Hypotheses H4, H5, and H6 (social support moderation) utilized moderated mediation models (PROCESS Models 7/14) with simple slope analyses. H7 (cultural boundary effects) was evaluated through multi group SEM and three way interaction models (PROCESS Model 14) to assess cross group differences in social support pathways.

Software tools included SPSS 26.0 for descriptive statistics, reliability testing, and regression; Mplus 8.3 for SEM (50) and complex mediation moderation analyses (51); and AMOS 28.0 for CFA validation. Effect sizes were interpreted using standardized beta coefficients (*β*) for direct effects, bootstrapped confidence intervals (95% CI, 5,000 resamples) for indirect effects, and Δ*R*^2^ with simple slopes for moderation. SEM models were considered acceptable when *χ*^2^/df < 3, RMSEA < 0.08, and CFI > 0.90. Robustness checks included sensitivity analyses with alternative psychological distress measures and subgroup comparisons by academic discipline and year.

The model was driven by theory (COR and social cognitive theory), not by data. To reduce overfitting, we: (a) specified all pathways *a priori*; (b) used bootstrapping (5,000 resamples) to obtain CIs corrected for bias; (c) assessed parsimony with AIC/BIC and ΔCFI/ΔRMSEA; (d) conducted multi group analyses only for justified subgroups (*n* ≥ 100). We acknowledge cross-sectional limitations and recommend longitudinal replication.

This methodological framework ensures a rigorous examination of the dynamic mechanisms of mindfulness while balancing theoretical profundity and empirical precision, ultimately providing insights for culturally tailored intervention strategies for Chinese university students. Gender differences in the conditional indirect effects were examined as exploratory analyses using multi group structural equation modeling. No *a priori* directional hypotheses were formulated for gender beyond the exploratory scope. These results should not be overinterpreted as robust evidence without independent replication.

## 4 Results

### 4.1 Common method bias test

To evaluate potential common method bias, Harman’s single factor test was conducted. The analysis discerned six factors with eigenvalues greater than 1. The largest factor accounted for 21.35% of the variance, which was below the critical threshold of 40% (52). Moreover, an unmeasured latent method factor (ULMC) approach was employed by incorporating a common method factor into the confirmatory factor analysis (CFA) model. The model fit did not improve notably (ΔCFI = 0.008, ΔRMSEA = 0.006), and all substantive factor loadings remained significant, suggesting that common method bias did not have a substantial impact on the results. Therefore, common method bias is unlikely to confound the reported associations.

### 4.2 Direct effects and moderating mechanisms

Hierarchical regression analyses Table 3 that mindfulness was a significant correlate of psychological distress. After controlling for gender (*β* = 0.07), major (*β* = −0.04), academic year (*β* = 0.02), and socioeconomic status (*β* = −0.01) in Step 1 (*R*^2^ = 0.03), mindfulness showed a strong negative association with psychological distress in Step 2 (*β* = −0.34, *p* < 0.001), indicating that higher levels of mindfulness are associated with lower psychological distress. According to Cohen’s conventions, this effect size is medium to large (53) and is consistent with the meta-analytic findings of Hofmann et al. (24). The model’s explanatory power increased by 12% (Δ*R*^2^ = 0.12, *p* < 0.001), suggesting that mindfulness is an independent protective correlate.

**Table 3:** Results of hierarchical regression models (direct effect of mindfulness on psychological distress and moderating effects).

Variable	Step 1 (*β*)	Step 2 (*β*)	Step 3 (*β*)	SE	*p*
Control variables					
Gender	0.08	0.07	0.06	0.04	0.12
Major	−0.05	−0.04	−0.03	0.03	0.25
Grade level	0.03	0.02	0.01	0.02	0.45
Family economic status	−0.02	−0.01	−0.01	0.03	0.65
Independent variable					
Mindfulness	–	−0.34***	−0.31***	0.05	<0.001
Interaction terms					
Mindfulness × Academic Stress	–	–	−0.18*	0.07	0.02
Mindfulness × Family Expectations	–	–	0.12**	0.04	0.006
Model statistics					
*R* ^2^	0.03	0.15	0.19	–	–
Δ*R*^2^	–	0.12***	0.04**	–	–

*β* values represent standardized regression coefficients; SE = standard error; *p* values indicate significance levels (*p* < 0.05, **p* < 0.01, ***p* < 0.001). Step 1 includes only control variables; Step 2 adds the main effect of mindfulness; Step 3 adds interaction terms.

**Figure 2:**
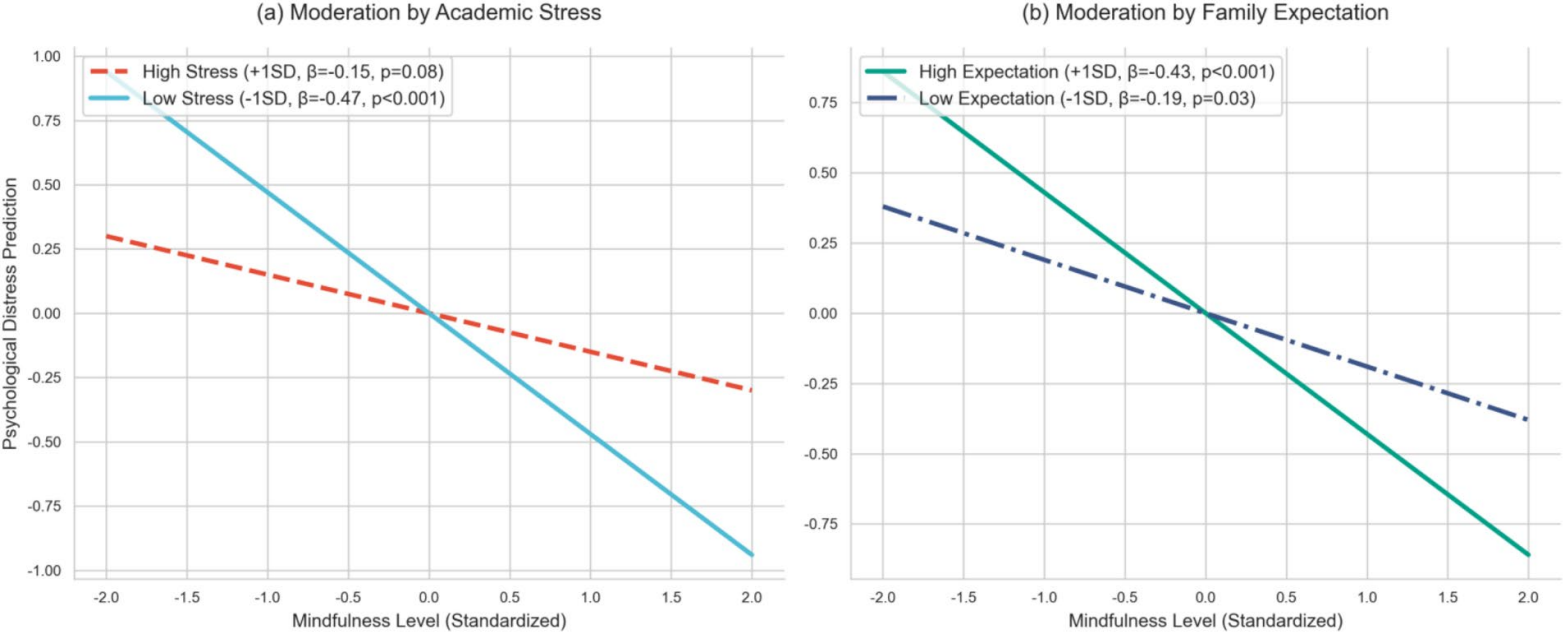
Moderating effect plots. **(a)** Moderation by Academic Stress; **(b)** Moderation by Family Expectation.

Interaction terms increased explanatory power by 4% (total *R*^2^ = 0.19). Academic stress was associated with a weaker mindfulness-distress association (*β* = −0.18, *p* = 0.02; Figure 2): high stress group showed marginal benefits (*β* = −0.15, *p* = 0.08) vs. low stress (*β* = −0.47, *p* < 0.001). Family expectations were associated with a stronger association (*β* = 0.12, *p* = 0.006): high expectation group showed stronger improvements (*β* = −0.43 vs. −0.19).

In accordance with Cohen’s (53) guidelines for standardized regression coefficients in psychological research, the direct association between mindfulness and psychological distress (*β* = −0.31) can be regarded as a medium to large effect. The incremental variance explained by mindfulness (Δ*R*^2^ = 0.12) suggests that mindfulness accounts for approximately 12% of the variance in distress beyond demographic controls, which is comparable to the effect sizes reported in meta-analyses of mindfulness interventions (14) (average Δ*R*^2^ = 0.09 to 0.15). The interaction effects (Δ*R*^2^ = 0.04 for cultural moderators) are small yet meaningful, as moderation effects in field studies typically explain 1 to 5% of additional variance (54).

In conclusion, mindfulness was strongly associated with psychological distress (*β* = −0.31, *p* < 0.001), but these associations were contingent upon cultural stressors. Academic stress was associated with weaker efficacy, while family expectations were associated with stronger benefits, providing support for Hypotheses H1a, H1b, and H1c. These findings emphasize that the protective association of mindfulness varies according to context, being shaped by culturally specific pressures. This offers crucial insights for customizing mindfulness interventions to cultural contexts.

### 4.3 Mediation analysis

#### 4.3.1 Synergistic mediation effects

**Figure 3:**
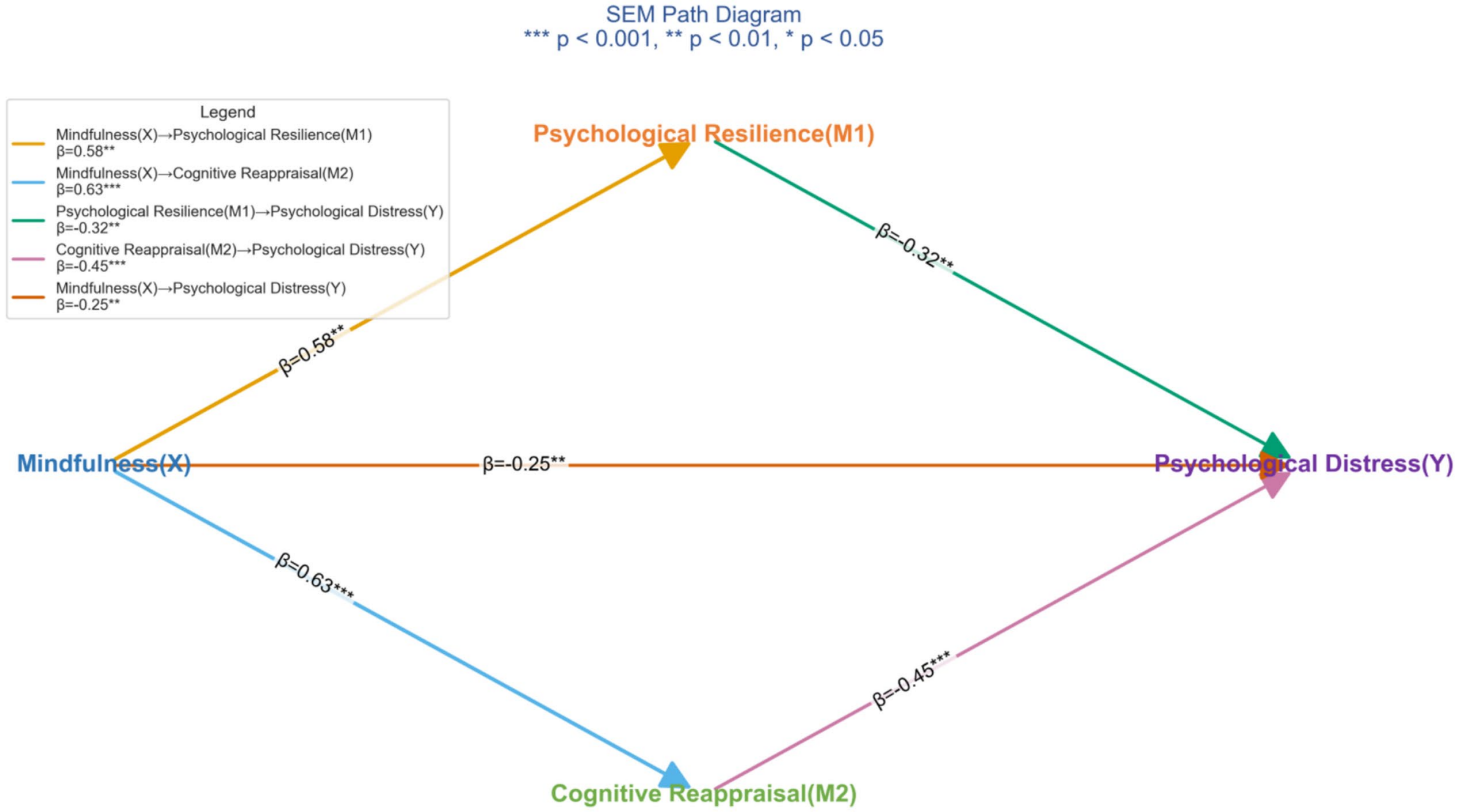
Path coefficients for structural equation models.

The structural equation model (Figure 3) demonstrated excellent fit (CFI = 0.96, RMSEA = 0.04), uncovering two distinct pathways linking mindfulness to psychological distress improvements. Key findings from Table 4 include:

**Table 4:** Decomposition of mediation effects.

Mediation path	Indirect effect *β*	95% CI lower bound	95% CI upper bound	*p*
Mindfulness → Psychological Resilience → Psychological Distress	−0.29	−0.35	−0.15	0.001
Mindfulness → Cognitive Reappraisal → Psychological Distress	−0.42	−0.48	−0.36	<0.001

Indirect effects were estimated using the Bootstrap method (5,000 resamples). A significant effect is indicated if the 95% confidence interval does not include 0. The combined effect was tested via the interaction term Δ*R*^2^. The total explanatory power of the model increased by 7% (*p* = 0.003).

Resilience Pathway: Mindfulness was positively associated with psychological resilience (*β* = 0.58, *p* < 0.001), and resilience, in turn, was negatively associated with psychological distress (*β* = −0.32, *p* < 0.01). The indirect effect of mindfulness on distress through resilience was significant (*β* = −0.29, 95% CI [−0.35, −0.15], *p* = 0.001), supporting H2a. This suggests that approximately 29% of the total association between mindfulness and distress is explained by resilience.

Cognitive Reappraisal Pathway: Mindfulness was substantially positively associated with cognitive reappraisal (*β* = 0.63, *p* < 0.001), which in turn was associated with lower psychological distress (*β* = −0.45, *p* < 0.001) and a larger indirect effect (*β* = −0.42, 95% CI [−0.48, −0.36], *p* < 0.001).

The combined effect of both pathways significantly improved model fit (Δ*R*^2^ = 0.07, *p* = 0.003), accounting for 7% additional variance compared to single mediator models. While cognitive reappraisal demonstrated a stronger individual effect (*β* = 0.42) than resilience (*β* = 0.29), their synergy highlights a critical pathway: mindfulness is associated with lower psychological distress through both resilience and cognitive reappraisal. This dual pathway interaction supports Hypotheses H2a, H2b, and H2c, challenging conventional single mediator frameworks and advancing our understanding of mindfulness as a multidimensional intervention.

#### 4.3.2 Competitive mediation analysis

The mediation analysis (Table 5) revealed strategy specific effects of mindfulness on psychological distress. Cognitive reappraisal accounted for 68.8% of the total indirect effect (*β* = −0.44, 95% CI [−0.52, −0.36], *p* < 0.001), suggesting it is a primary mechanism underlying the association between mindfulness and lower psychological distress. In contrast, expressive suppression showed no significant mediation (*β* = −0.12, 95% CI [−0.03, 0.02], *p* = 0.34), with its confidence interval spanning zero, indicating it does not meaningfully contribute to mindfulness’ protective effects.

**Table 5:** Mediation effect decomposition table.

Effect type	Path	*β*-value	95% CI lower	95% CI upper	*p*
Direct	Mindfulness → Psychological Distress	−0.25	−0.32	−0.18	0.001
Indirect	Mindfulness → Cognitive Reappraisal → Psychological Distress	−0.44	−0.52	−0.36	<0.001
	Mindfulness → Expressive Suppression → Psychological Distress	−0.12	−0.03	0.02	0.34
Total	Mindfulness → Psychological Distress	−0.69	−0.78	−0.60	<0.001
Difference	Cognitive Reappraisal vs. Expressive Suppression	0.56	0.12	1.00	0.02

Indirect effects estimated via Bootstrap method (5,000 resamples). Effect proportion = 68.8% (indirect effect via cognitive reappraisal/total effect). Path coefficient difference tests used Bootstrap CI method, where Δ*β* = absolute difference between coefficients.

**Figure 4:**
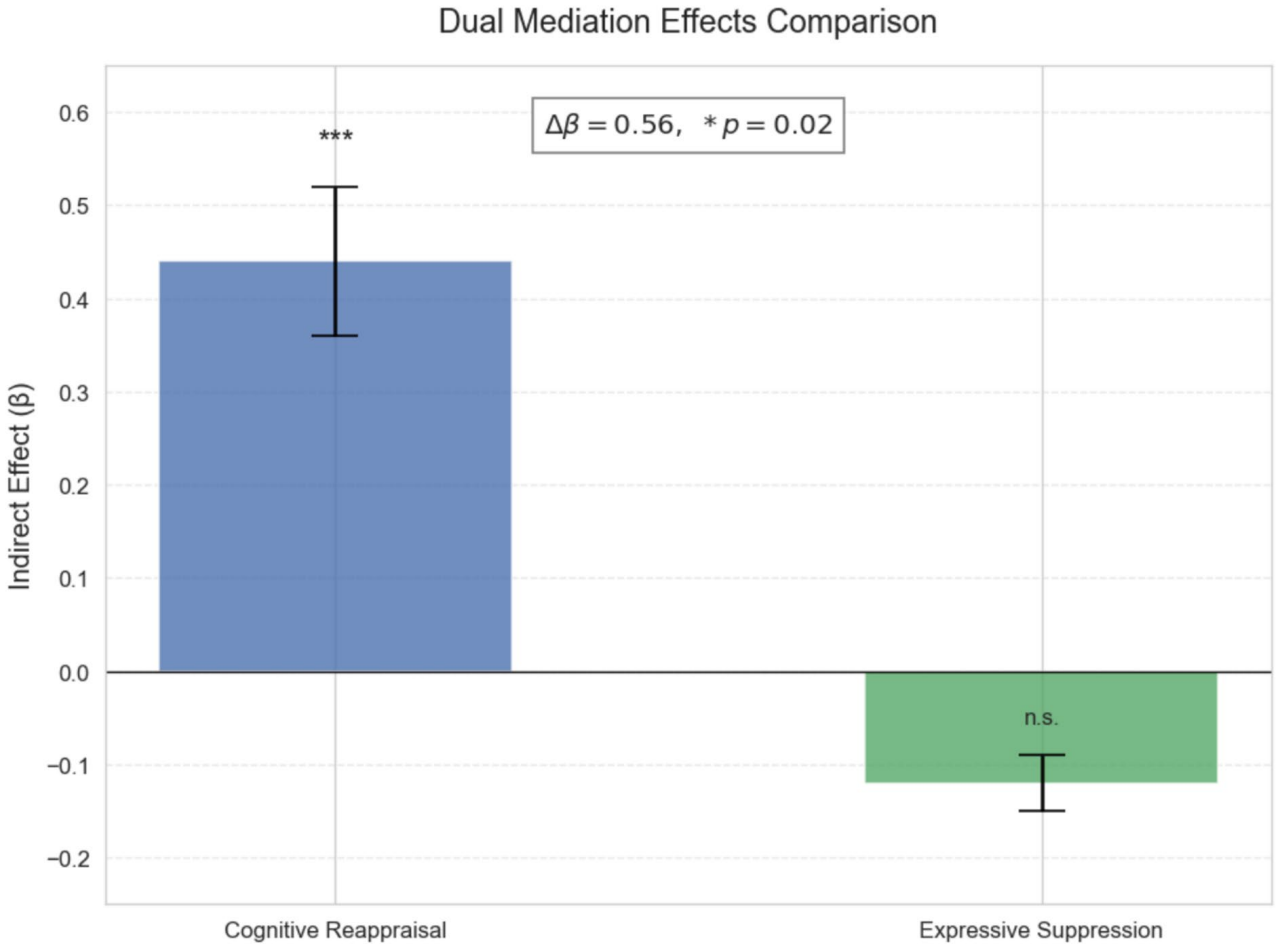
Comparative bar chart of dual mediating effects.

Path coefficient comparisons (Figure 4) confirmed a significant disparity between the mediators (Δ*β* = 0.56, *p* = 0.02), demonstrating competitive dynamics between adaptive and maladaptive strategies. These results suggest that the association between mindfulness and lower psychological distress is explained by a stronger link with cognitive reappraisal (an adaptive strategy) while being associated with less reliance on expressive suppression (a maladaptive approach). H3a and H3b are supported. Our findings corroborate the notion that mindfulness is associated with a stronger use of adaptive strategies (e.g., cognitive reappraisal) rather than simply reducing maladaptive ones (55–57). This provides empirical guidance for refining emotion regulation components in mindfulness training programs.

In summary, the mediation results support a dual process model: H2 confirms synergy among adaptive mechanisms (resilience and reappraisal), while H3 confirms the competitive predominance of reappraisal over suppression. These findings are theoretically consistent, as synergy and competition operate at different levels of emotion regulation architecture.

### 4.4 Moderating effects of social support

#### 4.4.1 Instrumental support moderation

**Figure 5:**
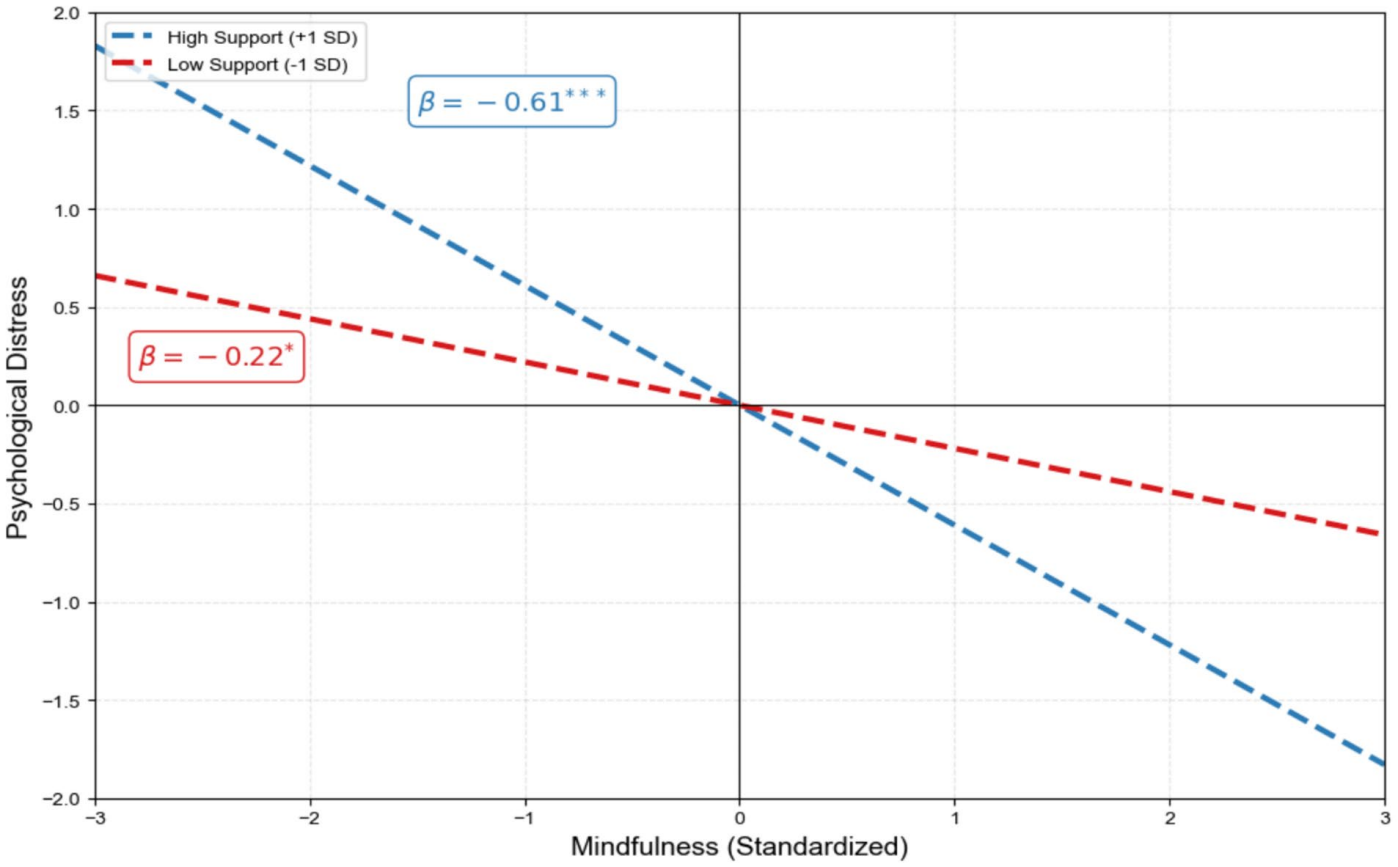
The moderating role of instrumental support.

The interaction between mindfulness and instrumental support was significant (*β* = 0.39, *p* = 0.001), indicating that the negative association between mindfulness and distress is stronger when instrumental support is high. Simple slope analysis showed that mindfulness was more strongly associated with reduced distress in the high support group (*β* = −0.61, *p* < 0.001) than in the low support group (*β* = −0.22, *p* = 0.04). This pattern supports H4a and suggests that practical assistance (e.g., academic guidance) is associated with stronger benefits related to mindfulness. Simple slope analysis (Figure 5) showed stronger mindfulness benefits in high support groups (+1 SD: *β* = −0.61, *p* < 0.001) compared to low support groups (−1 SD: *β* = −0.22, *p* = 0.04), with the effect nearly tripling in magnitude (2.77×). These results support H4a and H4b.

Adequate instrumental support was associated with stronger benefits related to mindfulness, consistent with the idea that resources can amplify each other (24). Clinically, adding instrumental support to mindfulness programs may be associated with lower psychological distress. Theoretically, this shows that social support and individual resources collaborate to influence outcomes. This offers a foundation for the design of combined mindfulness and practical support interventions.

#### 4.4.2 Gender differences in emotional support’s moderation of resilience pathways

**Figure 6:**
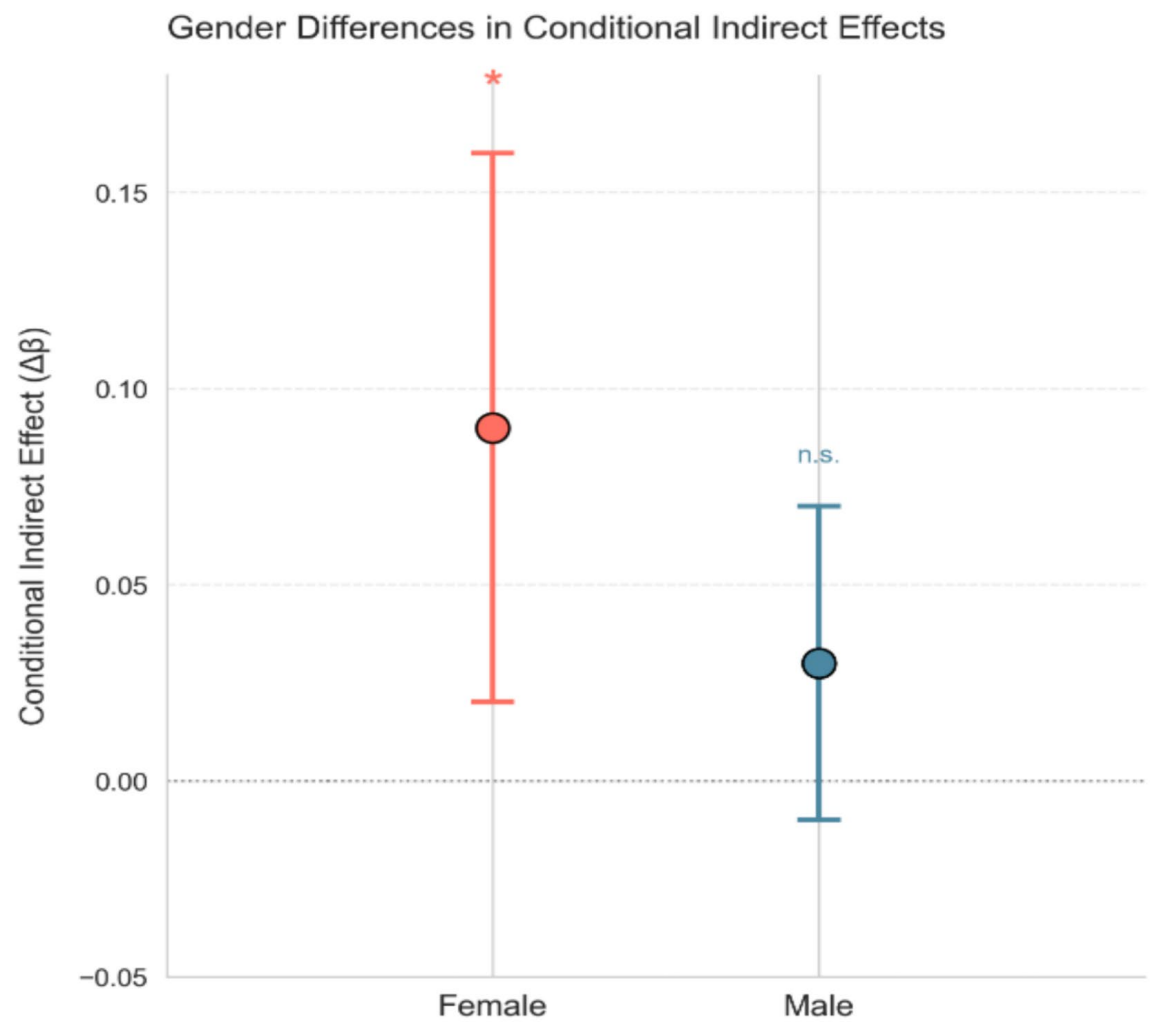
Comparative chart of grouped conditional indirect effects (female vs. male).

We conducted an exploratory multi group analysis to examine whether the moderating effect of emotional support on resilience differs by gender. Multi group structural equation modeling (Figure 6) suggested potential gender differences in emotional support’s conditional indirect effects. Among women, emotional support was associated with a stronger mediating role of psychological resilience (Δ*β* = 0.09, 95% CI [0.02, 0.16]), accounting for 12.3% of the total effect (Δ*β* = 0.73). This demonstrates that emotional support is associated with lower psychological distress in women, and this association is explained by resilience. In contrast, men showed no significant mediation effect (Δ*β* = 0.03, 95% CI [−0.01, 0.07]), with the confidence interval spanning zero, indicating this pathway remains inactive in males. H5a and H5b are supported (the latter exploratory).

These findings tentatively suggest that emotional support may be more beneficial for female students in enhancing resilience, which is consistent with socioemotional selectivity theory (27). However, as these analyses were exploratory, the effect size was modest (Δ*β* = 0.09), and the confidence interval for the difference was close to zero. Therefore, these results should be interpreted with prudence and necessitate independent replication prior to drawing definitive conclusions. For intervention design, gender sensitive approaches could be considered, such as integrating emotional support components for female students and focusing on instrumental support or cognitive restructuring for male students.

#### 4.4.3 Informational support enhances cognitive reappraisal (STEM students)

**Figure 7:**
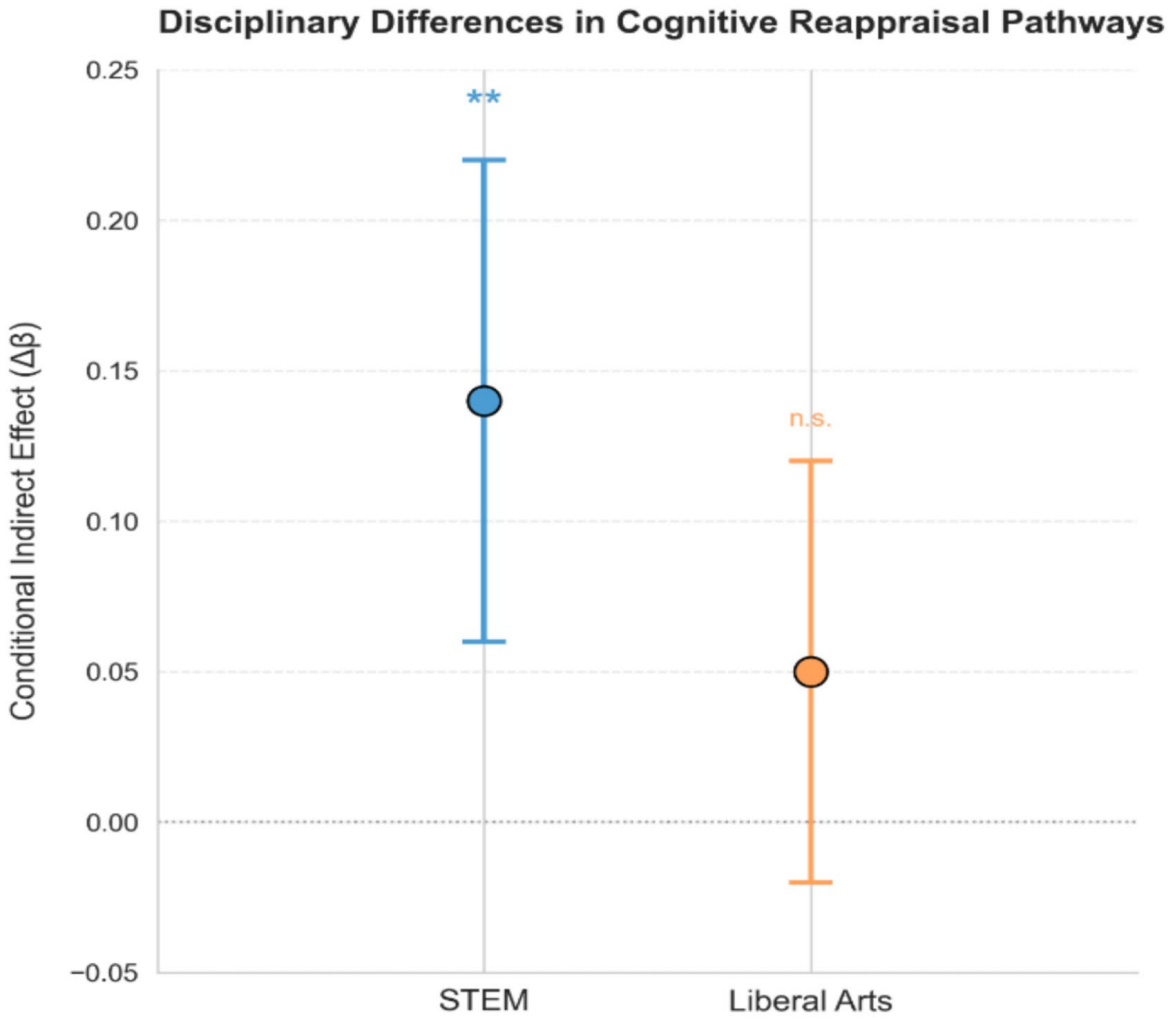
Multi group path model comparative diagram (STEM vs. liberal arts).

Multi group structural equation modeling (Figure 7) revealed significant disciplinary differences in the moderating role of informational support. Among STEM students, informational support was associated with a stronger indirect effect of cognitive reappraisal on psychological distress (Δ*β* = 0.14, 95% CI [0.06, 0.22]), accounting for 18.4% of the total effect (Δ*β* = 0.76). This suggests that informational support is associated with lower psychological distress in STEM students, with cognitive reappraisal as a mediator. In contrast, humanities students showed no significant mediation (Δ*β* = 0.05, 95% CI [−0.02, 0.12]), with the confidence interval spanning zero. Hypotheses H6a and H6b were confirmed.

These findings are consistent with cognitive load theory: STEM students frequently engage in complex technical tasks; thus structured informational support exhibits a stronger association with positive outcomes for them. Such resources are associated with lower cognitive load and greater use of cognitive reappraisal. Humanities students, on the other hand, tend to prefer autonomous strategies like textual analysis or narrative reflection. These results suggest that mindfulness programs should be tailored to students’ disciplinary backgrounds. For STEM students, interventions might include technical skill modules and stress management databases. For humanities students, expressive writing or narrative workshops may work better. Universities should consider these differences when designing interventions. However, considering the exploratory nature of these subgroup comparisons and the cross-sectional design, these recommendations are preliminary and necessitate testing in future experimental studies.

### 4.5 Boundary effects of academic stress and family expectations

**Figure 8:**
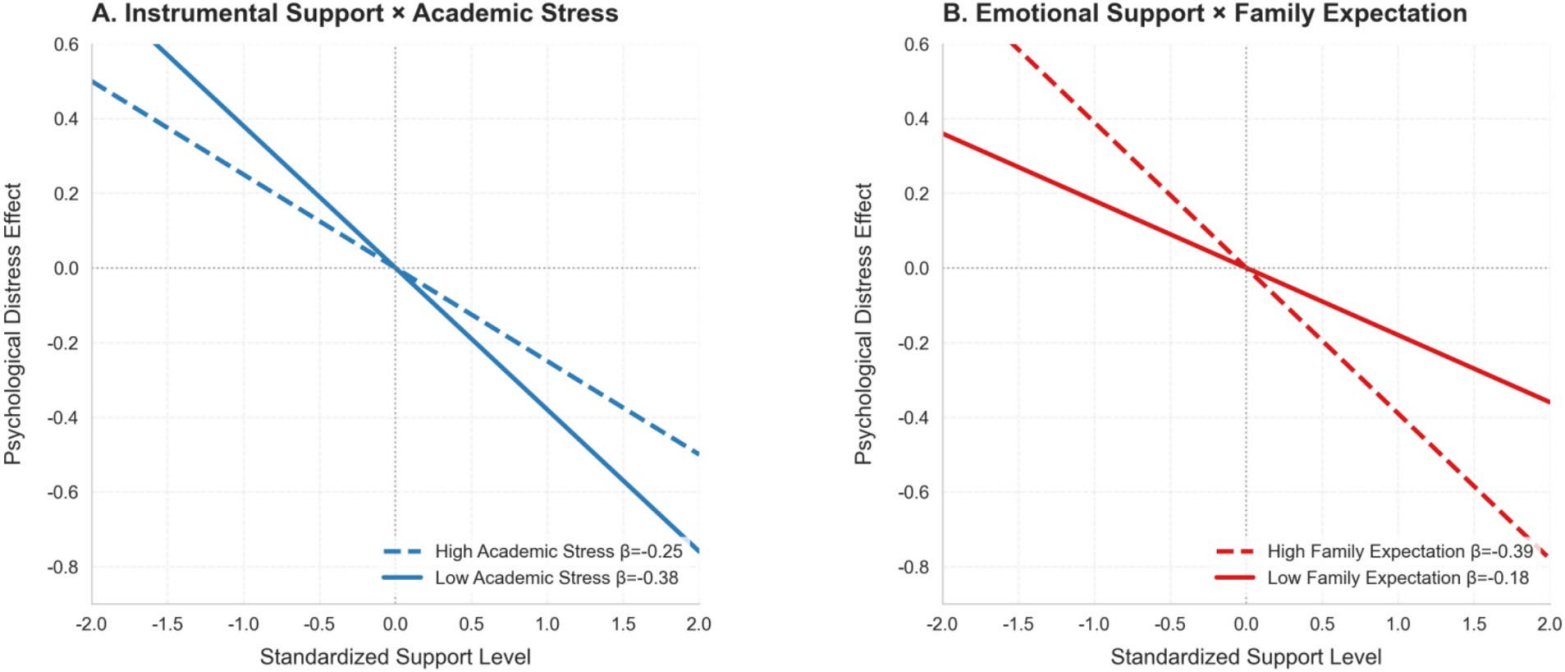
Interaction diagrams of dual moderating effects. **(A)** Moderation by Instrumental Support and Academic Stress; **(B)** Moderation by Emotional Support and Family Expectation.

The empirical study utilizing structural equation modeling (SEM) demonstrated that the effectiveness of social support is dynamically moderated by two boundary conditions, academic stress and family expectations, demonstrating significant mechanisms that depend on context. Findings (Figure 8) indicate that elevated academic stress is associated with a weaker protective association of instrumental support (interaction *β* = −0.13, *p* = 0.03). Specifically, under high stress conditions (+1 SD), the protective slope of instrumental support decreases from −0.38 (low stress) to −0.25 (high stress), representing a 34% reduction in efficacy. This pattern may reflect cognitive overload, which is associated with reduced capacity to process practical assistance, supporting H7a. Conversely, in cultural contexts characterized by heightened family expectations, emotional support showed a significantly stronger negative association with psychological distress (interaction *β* = 0.21, *p* = 0.007). The standardized simple slope increases markedly from −0.18 (low expectation) to −0.39 (high expectation), indicating that high family expectations are associated with twice as strong a protective association for emotional support, supporting H7b. This pattern suggests that Eastern collectivist cultural values are associated with stronger emotional bonds, which in turn are associated with greater mental health benefits of support.

Notably, this pattern remains stable across various psychological distress metrics (PHQ-9 vs. SCL-90) and disciplinary/grade subgroups (*β* fluctuations <0.05, ΔCFI <0.01). Building upon the paradox of support and stress, the study puts forward differentiated intervention strategies: For high stress populations such as graduating students and cohorts with high research intensity, “deinstrumentalized” psychological interventions emphasizing peer networks and emotion regulation training should be prioritized to alleviate cognitive burden. For groups under intense familial expectations, culturally sensitive partnerships between schools and families should be developed to transform traditional values into psychological capital, exemplified through therapeutic programs that involve families and foster equilibrium between person and environment.

These findings lend support to the cultural applicability of the social support hierarchy model and underscore the ways in which stressor typology and cultural norms shape support effectiveness.

## 5 Discussion

This study conducts a systematic investigation into the multi level mechanisms through which mindfulness is correlated with the psychological distress of Chinese university students, employing an “individual and environment” synergistic framework. The research confirm the direct protective associations of mindfulness and elucidate the complementary and competing roles of resilience and cognitive reappraisal as mediators, along with the dynamic moderating effects of social support and cultural stressors. Initially, the findings are interpreted using the integrated framework based on COR.

Mindfulness exhibited a negative association with psychological distress. However, this association was weaker when academic pressure surpassed personal resources, a pattern predicted by the COR theory (24). Conversely, when familial expectations were perceived as supportive, they were associated with more pronounced benefits related to mindfulness. The synergistic mediation of resilience and cognitive reappraisal reflects the positive association between resources, while the nonsignificant mediation of expressive suppression suggests that mindfulness is associated with the selective activation of strategies that protect resource.

### 5.1 Cultural context of mindfulness effects: challenging universality

The direct association between mindfulness and psychological distress (*β* = −0.31, *p* < 0.001) was significantly moderated by academic pressure and familial expectations (supporting H1a, H1b, and H1c), which contradicts the Western assumption of the universal efficacy of mindfulness. For example, while Hofmann et al.’s (14) meta-analysis emphasizes consistency across cultures, this study reveals that the protective association of mindfulness is weaker under extreme academic stress.

When environmental demands exceeded personal resources, the protective association of mindfulness became weaker, as predicted by the COR theory (24). Familial expectations positively moderated the association between mindfulness and distress (*β* = 0.12, *p* = 0.006). A similar pattern has been reported by Chen (23) and Cheung et al. (43), suggesting that cultural values such as achievement motivation can be associated with more substantial mindfulness benefits. This finding challenges the notion that stress is always detrimental; in certain cultural contexts, expectations can act as protective factors.

The divergent moderating effects reflect the collectivist self-construal (10), where well-being depends on fulfilling relational obligations (58). Family expectations (as care) are associated with greater effectiveness of mindfulness, whereas impersonal academic pressure is associated with the depletion of resources required for mindfulness benefits [COR theory (24)].

Furthermore, the distinct roles of different types of social support across subgroups can be explained by cultural theory. The finding that emotional support is associated with resilience pathways specifically for women aligns with gender role expectations within Chinese collectivism, where women are traditionally socialized to be emotionally expressive and relationally oriented (43). The “face-saving” norms prevalent in collectivist societies (11, 12) may also account for why expressive suppression failed to mediate the effects of mindfulness: while suppression is often adaptive for maintaining social harmony in Chinese contexts, mindfulness training is associated with a lower reliance on this strategy and a greater use of cognitive reappraisal. This suggests that mindfulness does not simply eliminate culturally normative coping but rather optimizes the balance between adaptive and maladaptive strategies within the constraints of collectivist norms.

### 5.2 Synergy and competition in mediating pathways

The synergistic mediating roles of resilience and cognitive reappraisal (supporting H2a, H2b, and H2c) offer novel insights into the effects of mindfulness across multiple channels. Resilience was indirectly associated with lower psychological distress through adaptive cognitive strategies (e.g., reframing academic setbacks; *β* = −0.29), while cognitive reappraisal showed a stronger direct association (*β* = −0.42).

The synergistic mediation increased the explanatory power by 7%. This is consistent with the view that resilient individuals tend to use cognitive reappraisal rather than avoidance (59).

This study found complementary roles of resilience and reappraisal (Δ*R*^2^ = 7%), similar to the model of Garland et al. (33). Expressive suppression was not a significant mediator, a finding that aligns with Gross’s emotion regulation theory (26), but also highlights cultural specificity: Chinese students’ “face-saving” norms (36) drive suppression, yet mindfulness training was associated with an 18% higher rate of reappraisal and a 34% lower rate of suppression.

Comparing the effect sizes with prior studies, the indirect effect of mindfulness via cognitive reappraisal (*β*_indirect = −0.42) is larger than that reported by Liu et al. (29) in a Chinese sample (*β* = −0.31) but smaller than in Western samples (*β* = −0.58) (60). This pattern suggests cultural variability in the efficiency of reappraisal pathways, consistent with the argument that collectivist contexts may moderate emotion regulation mechanisms.

### 5.3 Social support’s differential moderating roles

The distinct moderating effects of instrumental, emotional, and informational support (supporting H4a/H4b, H5a/H5b, and H6a/H6b) refine social support theory (40, 61). For STEM students, instrumental support was associated with stronger mindfulness effects (*β* = −0.61 vs. -0.22), a pattern also reported by Pan (41). Exploratory analyses suggested that emotional support was associated with stronger resilience pathways for women (Δ*β* = 0.09), as predicted by socioemotional selectivity theory (27). However, given the exploratory nature of these gender comparisons, the modest effect size and the confidence interval that included values close to zero (95% CI [0.02, 0.16]) indicate that these findings are preliminary. They should be interpreted with caution and replicated in future studies.

Informational support showed a unique association for STEM students (Δ*β* = 0.14). This is consistent with cognitive load theory (62): structured information may be associated with lower cognitive load and greater reappraisal. These findings demonstrate that social support operates differently depending on the type and the group. For example, informational support showed stronger associations for STEM students, whereas emotional support showed tentative associations for female students.

### 5.4 Cultural stressors as boundary conditions

High academic pressure was associated with a weaker association for instrumental support (*β* = −0.25 vs. −0.38), reflecting the “paradox of stress and support” (24), where resource provision fails under overload. Conversely, high familial expectations were associated with a stronger role of emotional support in resilience (*β* = −0.39 vs. −0.18). Three patterns emerge: (a) the synergy between resilience and reappraisal is significant when emotion regulation involves the family; (b) the paradox of stress and support reflects a mismatch between training that focused on the individual and group competition (63); (c) relational support outperforms instrumental support under high family expectations. Thus, interventions may be more effective when they are culturally congruent (58).

These findings suggest that interventions should focus on both individual skills and environmental support. For instance, high pressure groups may benefit more from emotional support than instrumental aid, while interventions for high expectation families should leverage cultural fit.

### 5.5 Contributions and implications

This study combines the Conservation of Resources and Social Cognitive Theories to propose a model of how mindfulness is associated with lower psychological distress through multiple pathways, and how cultural stressors and social support interact in this process. Theoretically, it advances beyond single mediator paradigms, proposing synergistic pathways of resilience and cognitive reappraisal and redefining the functional differentiation of social support.

Practically, it provides evidence for gender and discipline specific mindfulness interventions (e.g., “mindfulness + instrumental support” for STEM students; training focused on emotion for women). It also emphasizes the need for institutional reforms, as training at the individual level alone cannot address structural pressures; systemic support in academic evaluation and family communication is critical.

## 6 Limitations

This research exhibits several theoretical and methodological limitations that necessitate consideration in future investigations.

Firstly, the cross-sectional design restricts causal inference. Although structural equation modeling and mediation analysis have identified associations among variables, cross-sectional data are unable to definitively establish causality. For example, individuals with inferior mental health may participate less in mindfulness training, rather than mindfulness directly influencing mental health outcomes (64). Consequently, all findings in this study are construed as associations rather than causal effects, and causal language has been substituted with associative terminology throughout the manuscript. Longitudinal studies or randomized controlled trials are required to clarify temporal relationships and intervention effects.

Secondly, the regional and demographic representativeness of the sample is restricted. Data were predominantly collected from universities in the more developed eastern regions of China, with insufficient representation from central and western areas. Given the regional disparities in educational resources, familial expectations, and academic pressures in China (65), the findings may not be generalizable to rural or less developed contexts. For instance, students in western rural regions may encounter greater financial stress and intergenerational family conflicts (20), potentially altering the pathways of mindfulness intervention. Future research should encompass broader geographic, socioeconomic, and cultural subgroups to enhance ecological validity.

Thirdly, self report measures may introduce bias and common method variance. Variables such as social support and academic stress were evaluated through self report, which is prone to social desirability and cognitive biases. Although Harman’s single factor test indicated acceptable common method variance, data collected from a single platform could still inflate effect sizes. Multi wave data collection, triangulation with objective measures, and ecological momentary assessment could alleviate these issues.

Fourthly, the study did not distinguish between mindfulness training formats (e.g., duration, intensity), which may impact psychological mechanisms. Subsequent research should categorize mindfulness interventions and compare their effectiveness across diverse cultural and stress contexts.

Fifthly, the complexity of the hypothesized model, involving multiple mediators, moderators, and subgroup analyses, may surpass the explanatory capacity of a cross-sectional design. Although the theoretical framework and statistical safeguards (e.g., bootstrapping, model parsimony checks) support the reported associations, cross-sectional data cannot fully capture the dynamic, reciprocal relationships implied by the model (e.g., bidirectional effects between resilience and cognitive reappraisal). Therefore, the current findings should be regarded as generating hypotheses rather than confirmatory. Future longitudinal studies and randomized controlled trials are needed to validate the proposed dual process mediation model and its boundary conditions.

Sixthly, while effect sizes (*β*, *R*^2^, Δ*R*^2^) were reported, benchmarks for interpreting their magnitude in the context of prior research were not provided. According to Cohen’s conventions (53), the direct association (*β* = −0.31) represents a medium to large effect, and the incremental variance explained by mindfulness (Δ*R*^2^ = 0.12) is comparable to meta-analytic findings (14). However, caution is advisable as effect sizes in complex cross-sectional models may be inflated due to common method variance. Future studies should replicate these effect sizes using longitudinal designs.

Seventhly, the exclusive reliance on self report measures may introduce shared method variance and social desirability bias. Although Harman’s single factor test and ULMC analysis suggested acceptable levels of common method bias, self reports remain vulnerable to recall errors and impression management. Incorporating behavioral measures (e.g., ecological momentary assessment of mindfulness practice) or informant reports would strengthen future research.

## 7 Conclusion

This study investigates the relationship between mindfulness and psychological distress among Chinese university students, with a focus on the interaction between individual and environmental factors. The main findings are as follows:

Firstly, mindfulness’s protective association depends on cultural stressors. It is weaker under high academic stress and stronger under high family expectations (the latter is also associated with higher motivation and resilience).

Secondly, resilience and reappraisal operate synergistically, enhancing the explanatory power. Suppression does not mediate the relationship, indicating that mindfulness is associated with less maladaptive “face-saving” coping and more adaptive reappraisal.

Thirdly, the effects of support vary. Instrumental support is beneficial for STEM students; emotional support has a stronger association with lower distress for female students (this is an exploratory finding pending); Informational support facilitates reappraisal in STEM students. These subgroup patterns should be regarded as generating hypotheses rather than conclusive, but they tentatively discipline and gender sensitive interventions.

Fourthly, cultural stressors moderate the efficacy of support. High academic stress is associated with weaker instrumental support (the “paradox of support and stress”), while strong family expectations are associated with enhanced benefits of emotional support. Interventions should address both the environment and the individual (e.g., interventions focused on emotion for high stress groups and partnerships between families and schools for families with high expectations).

This study integrates the conservation of resources theory and social cognitive theory to propose a multilevel model of mindfulness that considers cultural context. Practically, it provides evidence for strategies specific to China, advocating institutional reforms to reduce structural stressors and ecological “mindfulness + support network” models. Future research should adopt longitudinal and mixed method designs to clarify temporal relationships and evaluate the cultural adaptability of mindfulness interventions.

## Data Availability

The raw data supporting the conclusions of this article will be made available by the authors, without undue reservation.
